# Nitric Acid-Treated Blue Coke-Based Activated Carbon’s Structural Characteristics and Its Application in Hexavalent Chromium-Containing Wastewater Treatment

**DOI:** 10.3390/molecules28247986

**Published:** 2023-12-07

**Authors:** Wencheng Wang, Hua Wang, Yunxuan Luoyang, Guotao Zhang, Xuchun Gao, Jian Li, Xia Li, Miao He

**Affiliations:** 1 College of Chemistry and Chemical Engineering, Yulin University, Yulin 719000, China; 2 Shaanxi Provincial Key Laboratory of Clean Utilization of Low-Modified Coal, Yulin University, Yulin 719000, China

**Keywords:** adsorption, Cr (VI), blue coke, potassium hydroxide activation, nitric acid modification

## Abstract

This study primarily focused on the efficient transformation of low-priced blue coke powder into a high-capacity adsorbent and aimed to address the pollution issue of hexavalent chromium (Cr (VI))-laden wastewater and to facilitate the effective utilization of blue coke powder. A two-step method was utilized to fabricate a blue coke-based nitric acid-modified material (LCN), and the impact of nitric acid modification on the material’s structure and its efficacy in treating Cr (VI)-contaminated wastewater was evaluated. Our experimental results illustrated that, under identical conditions, LCN exhibited superior performance for Cr (VI) treatment compared to the method employing only potassium hydroxide (LCK). The specific surface area and pore volume of LCN were 1.39 and 1.36 times greater than those of LCK, respectively. Further chemical composition analysis revealed that the functional group structure on the LCN surface was more conducive to Cr (VI) adsorption. The highest amount of Cr (VI) that LCN could bind was measured at 181.962 mg/g at 318 K. This was mostly due to chemisorption, which is dominated by redox reactions. The Cr (VI) removal process by LCN was identified to be a spontaneous, exothermic, and entropy-increasing process. Several tests on recycling and reuse showed that LCN is a stable and effective chromium-containing wastewater adsorbent, showing that it could be used in many situations.

## 1. Introduction

The topic of environmental protection has gained prominence as global economic integration progresses [1]. As a crucial industrial raw material, chromium is frequently released into natural water bodies as an industrial effluent. If huge amounts of untreated chromium-containing wastewater are released into the environment, it may seriously contaminate the environment [2,3]. Hexavalent and trivalent chromium are the two primary forms of chromium found in the natural environment [4,5]. Since Cr (VI) tends to build up in the human body and cause health issues such as skin irritation and lung cancer, managing chromium pollution primarily involves managing Cr (VI) [6]. Researchers have been actively developing a number of management techniques, including chemical reduction, adsorption, bioremediation, and electrocoagulation, to reduce the pollution caused by Cr (VI) [7]. Among these, the adsorption method has drawn a lot of interest because of its benefits, which include its high efficiency, adaptability, economy, and ease of use [8]. Cr (VI) removal is aided by carbon-based materials having large surface areas, well-developed pore structures, plenty of functional groups, and superior chemical qualities [9].

When considering sustainability and cost-effectiveness, there are a number of benefits to using cheap precursors for carbon-based products. Because of its advantageous qualities, blue coke, a byproduct of low-temperature coal pyrolysis, is widely used in a variety of applications [10]. Blue coke powder, which is produced during production and processing and has particle sizes less than 6 mm, negatively impacts the environment [10]. It is typically discarded or left unused. As a result, blue coke powder can be utilized as an easily accessible and affordable precursor for compounds based on carbon. Both surface chemistry and the physical or pore structure of carbon materials have a major impact on their adsorption capabilities [11].

One popular technique for raising the specific surface area of carbon compounds is activation. Potassium hydroxideis (KOH) is one of many activators that, because of its intercalation characteristics, can catalyze the gasification of precursors, encourage the creation of pores, and enhance the performance of adsorbents [12]. As a result, KOH is frequently used as an activator to improve the adsorption capacity of carbon compounds. In fact, our initial tests have shown that blue coke powder treated with KOH performs well for Cr (VI) [13]. Furthermore, the adsorption efficiency of carbon materials can be enhanced by adding functional group active sites to their surface [14]. Numerous surface functional groups can be produced by oxidative treatment [15]. Nitric acid oxidation is a commonly utilized method [16]. Strong acidic groups such as carboxyl groups are produced during nitric acid oxidation and can greatly enhance the adsorption capabilities of carbon materials [17].

Therefore, by integrating the changes in both pore structure and surface chemistry, in this study, we intended to make an adsorbent carbon material that has superior adsorption performance for Cr (VI). Our objective was to create an adsorbent with a high capacity for the adsorption of Cr (VI) and investigate the possibility of treating wastewater containing chromium with a blue coke adsorbent. This would enable the conversion of waste charcoal or low-value charcoal powder into high-value products.

## 2. Results and Discussion

### 2.1. Sample Characterization

**Figure 1:**
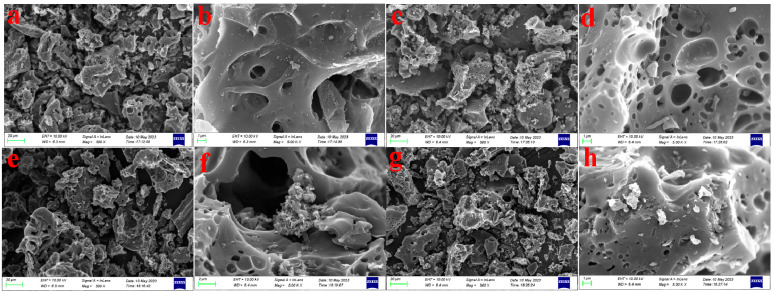
FE-SEM images: (**a**) LCK—500×; (**b**) LCK—5.00 K×; (**c**) LCN—500×; (**d**) LCN—5.00 K×; (**e**) LCK-Cr—500×; (**f**) LCK-Cr—5.00 K×; (**g**) LCN-Cr—500×; (**h**) LCN-Cr—5.00 K×.

The surface morphology of the samples was investigated using FE-SEM. It can be readily observed that, in comparison to LCK (Figure 1a,b), LCN (Figure 1c,d) exhibits a more developed porous structure with a smaller pore size and denser pore distribution after nitric acid treatment. Both LCK (Figure 1e,f) and LCN (Figure 1g,h) show noticeable agglomerates on the surface after chromium adsorption, and many pores display evident filling.

**Figure 2:**
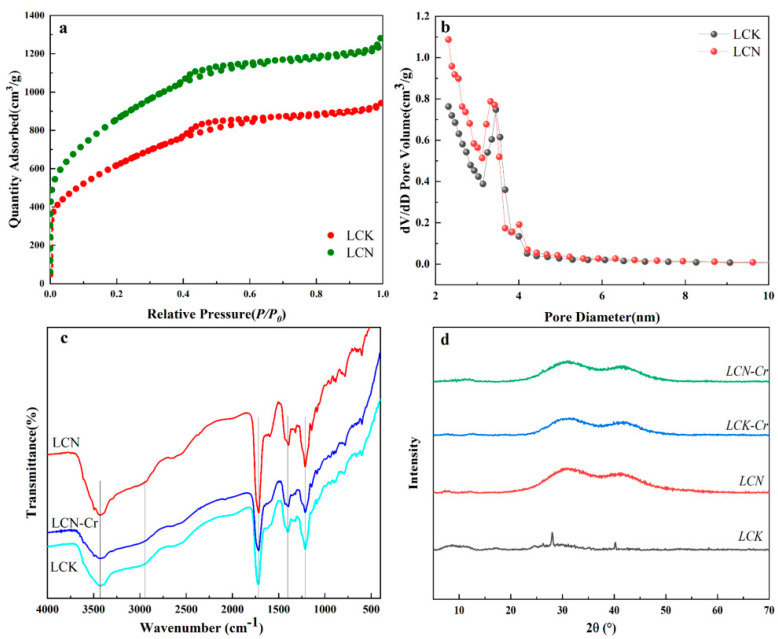
(**a**) The N_2_ adsorption and desorption curves of the samples; (**b**) the pore size distribution characteristics of the samples; (**c**) FTIR spectra of the samples; (**d**) XRD patterns of the samples.

An adsorbent-rich porous structure with a larger surface area is advantageous for enhancing the removal capacity of Cr (VI) [18]. The N_2_ adsorption and desorption isotherms of LCK and LCN are shown in Figure 2a. According to the IUPAC classification, at low relative pressures, the N_2_ adsorption and desorption isotherms of both materials resemble Type I isotherms, indicating a significant enhancement in adsorption capacity and the presence of a substantial number of micropores [19]. Hysteresis loops appear at a relative pressure of around 0.5, suggesting that both materials possess mesoporous structures [20]. When the relative pressure exceeds 0.9, the N_2_ adsorption–desorption curves for both materials continue to increase significantly, indicating the presence of some macropores [21]. The N_2_ adsorption and desorption curves for LCN are higher than those for LCK. This means that LCN has more pores than LCK. This is consistent with the FE-SEM analysis results presented above.

The pore size distribution characteristics of LCK and LCN are depicted in Figure 2b. It can be seen that the pore size distribution of LCK is similar to that of LCN, with the majority of pores having sizes below 4 nm. The porous structure parameters for both materials are summarized in Table S1. It is evident that LCN exhibits a surface area 1.38 times greater than that of LCK. Furthermore, the total pore volume of LCN surpasses that of LCK, even though the average pore size has decreased. This indicates that the modification with nitric acid does not disrupt the pore structure activated by potassium hydroxide, having no adverse impact on pore formation but rather enhancing the specific surface area and micropore volume.

The XRD diffraction peaks of LCK and LCN before and after adsorption are depicted in Figure 2d. It is evident that LCK exhibits distinct crystalline diffraction peaks before adsorption, whereas the diffraction peaks of LCN after nitric acid treatment transform into an amorphous pattern, likely attributed to the removal of inorganic crystalline components during the nitric acid washing process. Furthermore, the disappearance of peaks after adsorption in LCK indicates that the adsorption of Cr (VI) in strongly acidic solutions disrupts the crystalline structural composition of LCK.

The functional group distribution on the surfaces of LCK and LCN was investigated using FTIR spectroscopy (Figure 2c). It is evident that LCK and LCN exhibit roughly similar but locally distinct infrared spectra, indicating that the activation treatment altered the composition and quantity of surface functional groups in the materials. The peaks around 3420 cm^−1^ are attributed to the stretching vibrations of O-H bonds [22]. LCN shows a higher intensity peak at this position compared to LCK, suggesting that nitric acid activation enhances the number of hydroxyl functional groups on the material’s surface. However, the intensity of this peak decreases for LCN-Cr after adsorption, indicating the involvement of hydroxyl groups in the adsorption process of Cr (VI). The peaks in the range of 1720–1320 cm^−1^ are attributed to the vibrations of C=C skeletal bonds or C=O stretching vibrations [23,24,25]. Within this range, all three samples exhibit notable absorption peaks, with LCN displaying a higher peak intensity compared to LCK and LCN-Cr. This further suggests that nitric acid modification enriches the oxygen-containing functional groups and that these oxygen-containing functional groups are involved in the adsorption process [26].

### 2.2. Adsorptive Property

**Figure 3:**
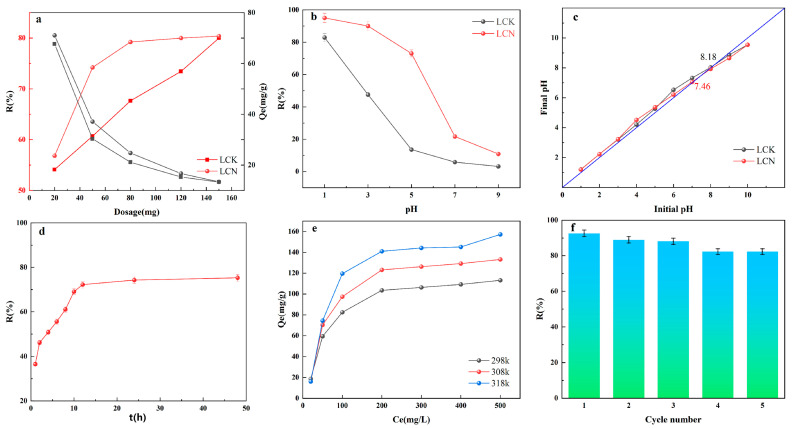
(**a**) Influence of sample dosage on Cr (VI) removal capacity and the adsorption amount of Cr (VI). (**b**) Impact of solution pH on Cr (VI) removal capacity. (**c**) Relationship between initial pH and final pH. (**d**) Effect of adsorption time on Cr (VI) removal capacity for LCN. (**e**) Influence of initial Cr (VI) concentration on Cr (VI) removal capacity and adsorption amount of Cr (VI) for LCN. (**f**) Effect of the number of regeneration cycles on Cr (VI) removal capacity for LCN.

The impact of LCK and LCN dosages on the removal of Cr (VI) and adsorption amount of Cr (VI) is depicted in Figure 3a. It can be observed that as the dosage increases, the removal efficiency of Cr (VI) steadily rises. At a dosage of 150 mg, both LCN and LCK exhibit removal efficiencies exceeding 80%, with no significant difference between them. To the contrary, when the dose is less than 150 mg, LCN consistently removes more than LCK, which suggests that LCN has more developed active sites for adsorbing Cr (VI) than LCK. We can guess that the large number of active sites in LCN is due to its well-developed microporous structure and the presence of many different oxygen-containing functional groups from the characterization studies of the above materials. The removal efficiency of Cr (VI) by both LCN and LCK shows a pattern of an initial rapid increase followed by a gradual decrease. This shows that competitive adsorption becomes more important as the number of active surface sites on the adsorbents increases [27]. 

From Figure 3a, it is evident that the amount of Cr (VI) adsorbed per unit mass of adsorbent decreases as the adsorbent dosage increases. This trend suggests that providing an excess of active sites while enhancing the removal of Cr (VI) from the solution reduces the efficiency of active site utilization on the adsorbent. The primary reason for the decline in active site utilization at higher adsorbent dosages is the reduction in adsorption capacity. This underlying issue may be attributed to the propensity for adsorbent particles to aggregate or remain at a significant number of adsorption sites due to excessive adsorbent usage [28]. The research results indicate that although increasing the adsorbent dosage can enhance Cr (VI) removal efficiency, it can also lead to inefficient adsorbent utilization. Through an economic evaluation, a dosage of 1 g/L was determined to be the ideal quantity for LCN.

The pH of the solution changes both the surface charge of the adsorbent and the level of protonation of functional groups [29]. The effect of pH on the removal efficiency of Cr (VI) by LCK and LCN was examined, as illustrated in Figure 3b. Both materials displayed a noticeable decline in Cr (VI) removal efficiency with increasing pH, particularly within the pH range of 1–7. Within this range, the removal efficiency of LCK and LCN decreased from 82.93% and 95.06% to 5.86% and 21.69%, respectively.

Results from the zero-point charge measurements for both materials are presented in Figure 3c, with LCK being measured at 7.46 and LCN at 8.18. When the pH of the solution is lower than the material’s zero-point charge, the sample’s surface gets protonated and becomes positively charged [21]. This leads to an electrostatic attraction between the anionic Cr (VI) and the positively charged sample surface, especially when the pH is below the material’s zero-point charge [30]. The stronger the positive charge on the sample’s surface, the better it can trap the chromium anions [31]. Regardless, when the pH goes above 7, there are more hydroxide ions, which makes it harder for the Cr (VI) anions to find a place to attach [32].

Additionally, researchers have found that the adsorbent takes in a lot of H^+^ ions when it removes Cr (VI), but this absorption decreases as the pH level increases [33]. When there are plenty of H^+^ ions around, Cr (VI) is more likely to be transformed into Cr (III) [21]. So, what we observed in this experiment is that as the pH level rises, the efficiency of Cr (VI) removal drops significantly due to a combination of oxidation–reduction and electrostatic attraction effects. In highly acidic solutions, the oxidation–reduction process becomes more dominant than electrostatic attraction. Based on these findings, a pH of 2 was chosen for subsequent experiments to further explore how well LCN removes Cr (VI) and the underlying mechanisms.

The regenerative capacity of adsorbents bears paramount significance within the domains of environmental conservation, resource stewardship, and economic efficacy. It serves to curtail costs, alleviate environmental footprints, and bolster the sustainability of industrial processes. Consequently, the regenerative efficacy of adsorbents typically ranks as a pivotal criterion in assessing their suitability for industrial applications and their intrinsic value. In this experimental setup, the regeneration of LCN was carried out by employing a sodium hydroxide (NaOH) solution. This choice was made because NaOH is alkaline, and it is known that this can break the bonds between the adsorbent and Cr (VI) anions [34]. From the first regeneration cycle to the last, the removal efficiency of LCN for Cr (VI) slowly decreased, reaching 92.59%, 88.98%, 88.12%, 82.35%, and 82.35% in each cycle, as shown in Figure 3f. After undergoing five regeneration cycles, the removal capacity decreased from the initial 92.59% to 82.35%. Despite this reduction, a substantial fraction of active sites was successfully restored, resulting in a sustained high capacity for Cr (VI) removal. Also, the small difference in how well Cr (VI) was removed between the fourth and fifth cycles after regeneration suggests that LCN may still be very good at removing Cr (VI) even after many cycles. LCN exhibits commendable reusability and is thus considered a promising and effective adsorbent for Cr (VI) removal.

### 2.3. Impact of Contact Time and Kinetic Analysis

The influence of adsorption time on the removal efficiency of Cr (VI) is depicted in Figure 3d. During the initial stage (0–4 h), LCN exhibits a significant enhancement in the removal rate of Cr (VI), suggesting the presence of abundant active sites on LCN at this juncture, with the majority of adsorption taking place on the external surface of the adsorbent [35]. Physical adsorption plays a pivotal role during this phase [36]. As the adsorption time increases, there is a notable deceleration in the rate of removal (4–10 h), indicating that as the active sites on the external surface diminish, the sample enters a sluggish adsorption phase, with a substantial portion of Cr (VI) infiltrating the pore channels and adsorbing onto the active sites on the internal surface. Chromium ions have to travel a longer distance through the pore channels because the internal surface has higher adsorption resistance than the external surface. This means that the ions have to take more time to reach the active sites [37]. As the adsorption time goes on, the active sites on the outside of the surface become less active. Instead, most of the adsorption happens on the inside, which slows down the removal rate even more. When the active sites on the internal surface are likewise exhausted, adsorption ultimately reaches an equilibrium state [38].

**Figure 4:**
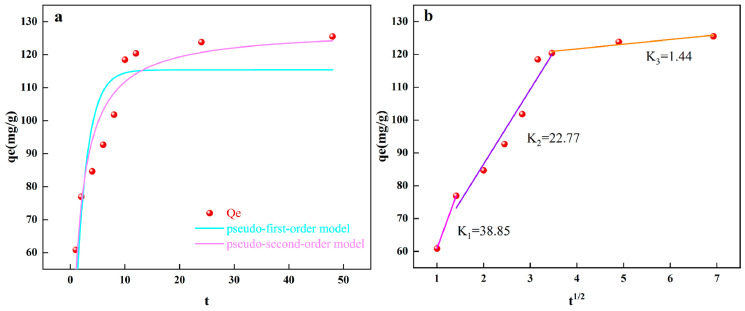
(**a**) The fitting curves of the pseudo-first-order model and pseudo-second-order model for kinetic analysis for LCN. (**b**) Fitting curves for intra-particle diffusion analysis for LCN.

By looking into the adsorption kinetics of LCN, we can learn more about how LCN and Cr (VI) stick together and figure out what controls the rates of Cr (VI) adsorption. By fitting adsorption kinetics, we can get a better idea of how the adsorption process changes over time, which leads to more accurate data that can be used in real life. The pseudo-first-order kinetic model interprets the adsorption process as the physical adsorption of a monolayer, where the concentration of Cr (VI) ions in the solution governs the adsorption rate [39]. The pseudo-second-order kinetic model describes adsorption rates between the adsorbent and adsorbate as being controlled by chemical adsorption processes [40]. By employing these two kinetic models (Supplementary S3), we investigate the adsorption behavior of LCN towards Cr (VI). The fitting curves are shown in Figure 4a. It is evident that, compared to the pseudo-first-order kinetic model, the pseudo-second-order kinetic fitting demonstrates superior performance with a higher R^2^ value (Table S2), suggesting that the adsorption of Cr (VI) by LCN is primarily governed by chemical adsorption rather than surface complexation and precipitation, thus emphasizing the role of surface chemical reactions in the removal of Cr (VI) by LCN [41].

A more in-depth investigation into the control mechanisms of adsorption rates was conducted using the intra-particle diffusion model (Supplementary S1). The results are presented in Figure 4b. It can be observed that none of the three adsorption trendlines pass through the origin, indicating a multi-step controlled adsorption process [42]. The intra-particle diffusion model divides the adsorption process into three stages: surface external diffusion, intra-particle diffusion, and surface adsorption. The order of rate constants for these three stages is as follows: k1 > k2 > k3, suggesting that as the contact time increased, the removal of Cr (VI) by LCN exhibited a diminishing trend, eventually attaining equilibrium. Many functional groups on the outside of LCN play a key role in the adsorption process, as shown by the short duration of the first stage and the large increase in adsorption [43].

### 2.4. Effect of Concentration and Adsorption Isotherm

One of the key parameters influencing the adsorbent’s removal capacity is the initial ion concentration. The impact of different initial concentrations (20–500 mg/L) on LCN’s adsorption of Cr (VI) was assessed (Figure 3e). It is evident that as the initial concentration of Cr (VI) increases, the equilibrium adsorption capacity of LCN for Cr (VI) also increases. In particular, at a starting concentration of 200 mg/L of Cr (VI), the equilibrium adsorption capacity of LCN shows a clear increase as the ion concentration rises. However, beyond this concentration, the rate of the increase in equilibrium adsorption capacity markedly diminishes. These results indicate that the equilibrium adsorption capacity of the adsorbent is sensitive to the initial concentration of the target pollutant. Higher starting concentrations make it so that surface adsorption sites are full during the first stage of adsorption, which makes bulk adsorption sites less available [44]. In contrast, at lower concentrations, the availability of bulk adsorption sites leads to a significantly higher removal efficiency.

**Figure 5:**
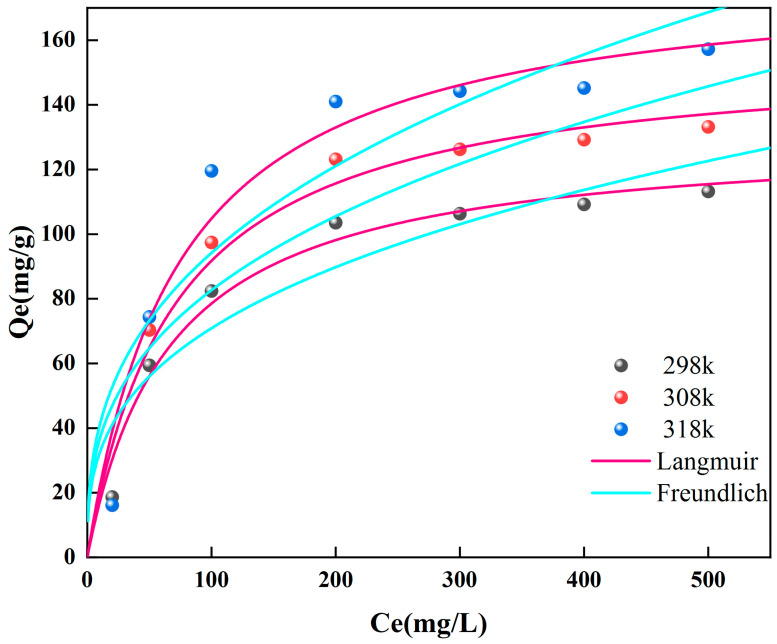
Adsorption isotherm fitting curves for LCN.

The adsorption isotherm modes (Supplementary S2) represent the relationship between the concentrations of solute molecules in two phases when the adsorption process reaches equilibrium at a specific temperature. Figure 5 shows the results of using the Langmuir and Freundlich models to analyze the data. Table S3 shows the relevant parameters of the fitted curves.

The Langmuir model says that the surface of the adsorbent has many even adsorption sites that are all equally attracted to the adsorbate and that the occupancy of one active site does not change the occupancy of nearby active sites [45]. The Freundlich model, on the other hand, is based on the idea that different surfaces adsorb different substances differently. It says that as active sites get more occupied, binding strength decreases [46]. Based on Figure 5, it is clear that the Langmuir model better describes how the molecules stick to LCN, showing that it happens on a flat, single-layer surface. 

The adsorbent’s maximum adsorption capacity is a key measure of its ability to adsorb. It is affected by things like the pore structure, the chemical properties of the surface, and the size and shape of the molecules that are adsorbed. In practical applications, the maximum adsorption capacity can be used to infer the adsorption capacity and service life of the adsorbent.

Table 1 shows that the Langmuir model says that LCN can adsorb up to 181.962 mg/g at 318 K, which is more than most carbon-based adsorbents. This suggests that LCN has significant potential for addressing environmental contamination by Cr (VI).

### 2.5. Temperature and Adsorption Thermodynamics

Temperature is a primary determinant of whether an adsorption reaction can occur, directly impacting both the adsorption capacity and rate of the adsorbent. Therefore, investigating the temperature effect is essential. Experiments for the removal of Cr (VI) were conducted under temperature conditions of 298, 308, and 318 K, with a focus on examining the influence of temperature on the adsorption performance. As shown in Figure 5, under otherwise identical conditions, the adsorption of Cr (VI) increases with increasing temperature. 

The study of adsorption thermodynamics (Supplementary S3) provides insights into the extent and driving forces of the adsorption process and allows for a comprehensive analysis of the reasons behind various factors affecting adsorption. By calculating the thermodynamic parameters of LCN’s adsorption of Cr (VI), the results are presented in Table S4. A negative ΔG indicates that the reaction is spontaneous, while a positive ΔH confirms that the adsorption process is endothermic, and a positive ΔS implies an increase in system disorderliness [50]. As shown in Table S4, ΔH is positive for the adsorption of Cr (VI). This means that Cr (VI) and the adsorbent are interacting in an endothermic way, meaning that the adsorption is increasing as the temperature rises. Also, ΔS > 0 means that LCN and chromium ions like each other [51], which is in line with previous studies on Cr (VI) adsorption [52]. Furthermore, ΔG < 0 suggests that the adsorption of Cr (VI) by LCN is spontaneous. As the temperature rises, the endothermic nature (ΔH > 0) and the increase in randomness at the reaction interface (ΔS > 0) both work in favor of getting rid of Cr (VI).

### 2.6. Adsorbent Mechanism

**Figure 6:**
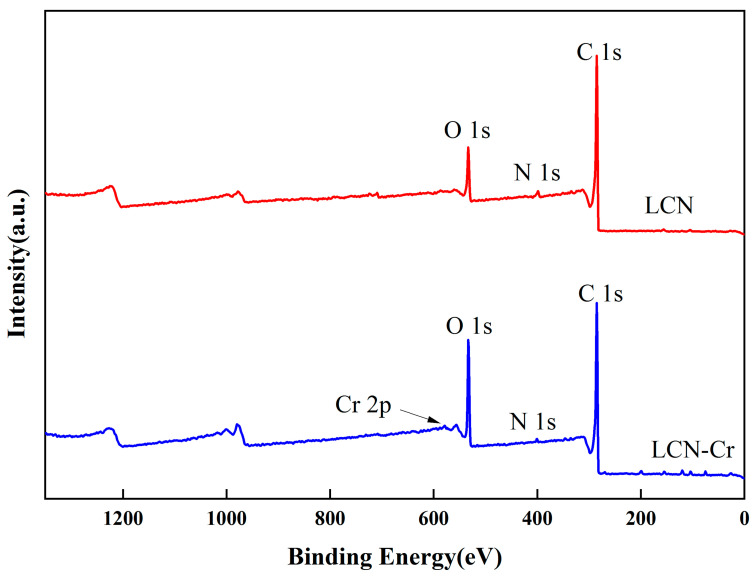
Full XPS spectrum of the samples.

XPS was used to examine the samples before and after Cr (VI) removal reactions in order to learn more about how the surface active groups of LCN and Cr (VI) interact with each other. The results of full-spectrum scans are presented in Figure 6. After adding Cr (VI), new characteristic peaks of Cr 2p showed up on LCN, along with the main characteristic peaks of C 1s, O 1s, and N 1s. These results indicate that chromium has adsorbed onto the surface of LCN, consistent with the SEM findings.

**Figure 7:**
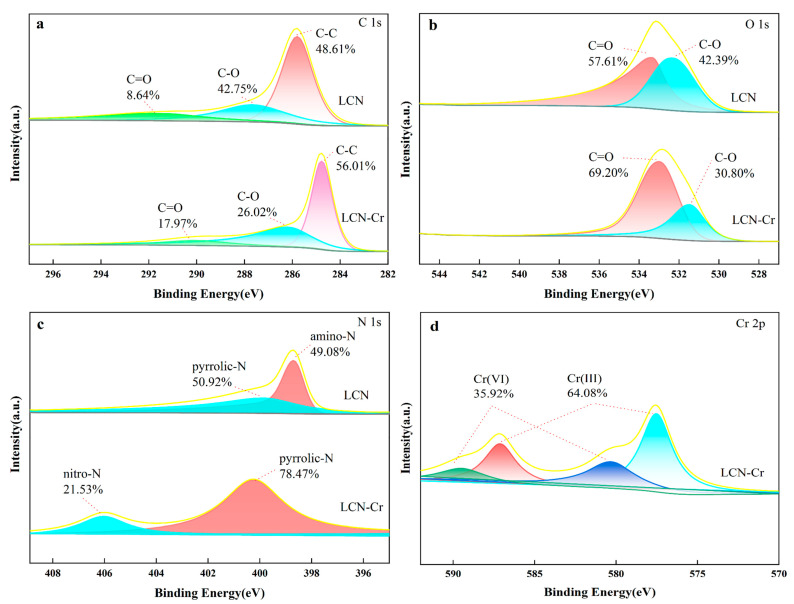
High-resolution XPS spectra of (**a**) C 1s, (**b**) O 1s, (**c**) N 1s, and (**d**) Cr 2p for LCN before and after adsorption.

Figure 7b displays the high-resolution, narrow spectrum of O 1s before and after adsorption. It is evident that in the curve of LCN before adsorption, the peak at 533.34 eV can be attributed to C=O, while the peak at 532.29 eV can be attributed to C-OH [53]. A comparison of the curves before and after adsorption reveals a noticeable shift and reduction in the peak intensity of C-OH, suggesting the involvement of OH groups in electron sharing during the adsorption process. This is consistent with the results obtained from the infrared analysis mentioned above.

Figure 7c presents the high-resolution narrow spectrum of N 1s before and after adsorption. There were two kinds of nitrogen species seen in the LCN curve before it was adsorbed: amino-N and pyrrolic-N [54]. The peak intensity of the pyrrolic-N group changes a lot after Cr (VI) adsorption. The peak intensity of the amino-N group goes away, and nitro-N shows up. This indicates the presence of potential electron transfer between the N-containing groups and Cr (VI).

Figure 7d shows four peaks corresponding to the characteristic peaks of Cr 2p 1/2 and Cr 2p 3/2, respectively. These peaks align with the characteristics of Cr (VI) and Cr (III), suggesting the coexistence of Cr (VI) and Cr (III) on the adsorbent material surface [53]. Under acidic conditions, Cr (VI) primarily exists as HCrO^4−^, and protonated groups such as hydroxyl and nitrogen-containing functional groups may oxidize it to Cr (III). Therefore, redox reactions may be one of the reasons for the simultaneous presence of Cr (VI) and Cr (III) on the adsorbent material. 

Based on the XPS analysis of LCN before and after Cr (VI) adsorption, it can be inferred that under acidic conditions, a small amount of Cr (VI) is reduced to Cr (III) by hydroxyl and amino groups, while some Cr (VI) interacts with hydroxyl and nitrogen-containing functional groups through electron transfer and electron sharing. Additionally, under low pH conditions, negatively charged Cr (VI) can be adsorbed by positively charged groups through electrostatic attraction. Thus, redox reactions, electron transfer, electron sharing, and electrostatic adsorption all contribute to the adsorption process of Cr (VI) on LCN.

## 3. Materials and Methods

### 3.1. Materials

The powdery blue coke raw material was sourced from BeiYuan Chemical Industry located in Yulin, Shaanxi, China. All reagents used in the experiment were purchased from Tianjin Zhiyuan Chemical Reagents Co., Ltd. (Tianjin, China), and were of analytical purity, with no additional processing taking place.

### 3.2. Sorbent Preparation

#### 3.2.1. The Preparation of Alkali-Modified Blue Coke

Take a specified amount of blue coke powder, pass it through a 200-mesh sieve, wash it multiple times with distilled water, and then dry it in a forced-air drying oven at 105 °C until a constant weight is achieved. The resulting sample was labeled as LC. Take LC and heat it up at 800 °C in a nitrogen atmosphere with a 1:4 ratio of blue coke powder to potassium hydroxide. Do this for 2 h at a rate of 5 °C/min and 100 mL/min of nitrogen flow. After pyrolysis, grind the sample, wash it three times with dilute hydrochloric acid, and rinse it with deionized water until the pH of the filtrate approaches neutrality. Subsequently, vacuum-filter it and dry it in a 105 °C drying oven until a constant weight is reached to obtain a sample labeled LCK.

#### 3.2.2. The Preparation of the Blue Coke Powder through a Two-Step Acid-Base Modification

Weigh 5 g of LCK and immerse it in a 30% HNO_3_ solution with a volume of 100 mL; stir magnetically for 6 h. Then, repeatedly wash the immersed material with deionized water until the filtrate reaches neutrality. After vacuum filtration, dry it overnight at 105 °C to obtain a modified carbon material (labeled LCN).

### 3.3. Material Characterization

The specific surface area, pore structure, and average pore size of the composites were assessed by conducting N_2_ adsorption–desorption isotherms at 77 K using a pore size analyzer (BET, ASAP 260, Micromeritics Instrument Corp., Norcross, GA, USA). To examine the surface morphology of the samples, a field emission scanning electron microscope (FE-SEM, SIGMA 300, Oberkochen, Germany) was employed. The functional groups of the materials were characterized using Fourier-transform infrared spectroscopy (FTIR, TENSOR 27, Bruker, Billerica, Germany). X-ray photoelectron spectroscopy (XPS, Thermo Scientific K-Alpha, Waltham, MA, USA) was used to find out what elements were in the materials before and after Cr (VI) adsorption. Additionally, the point of zero charge (PZC) was determined using the pH drift method (Supplementary Method S1). The concentrations of Cr (VI) were measured using a UV-vis spectrophotometer (UV-722S, Shanghai Lengguang Technology Co., Ltd., Shanghai, China).

### 3.4. Adsorption Experiments

Preparation of Cr (VI) Mother Liquor: Dissolve 1.4145 g of potassium chromate in 1 L of deionized water. Dilute as necessary for the experiment. Determine the concentration of Cr (VI) using the diphenylcarbazide spectrophotometric method. Add 20 to 150 mg of LCK or LCN to a 50-mL solution containing 50 mg/L Cr (VI). After constant shaking at 298 K for 6 h, measure the Cr (VI) content. Using a 50 mL solution containing 50 mg/L Cr (VI), add 0.1 g of the sample and investigate the effect of pH (2–10) on Cr (VI) removal. For the kinetic experiments, add 0.15 g of LCN to a 250 mL solution containing 100 mg/L Cr (VI) at pH 2 and sample at intervals from 0 to 48 h. Nine supernatants were collected, each with a volume not exceeding 1 mL. To assess the adsorption isotherms and thermodynamics, add 50 mg of the sample to a 50 mL solution at pH 2 under temperatures of 298 K, 308 K, and 318 K and Cr (VI) concentrations ranging from 20 to 500 mg/L. 

### 3.5. Material Reusability Experiment

Use a 0.05 mol/L sodium hydroxide solution to regenerate LCK and LCN after they are saturated with adsorbed Cr (VI). Evaluate the reusability of LCN using a repeated adsorption and desorption process. Here are the specific steps: In an acidic environment (pH = 2) at 298 K, add 20 mg of adsorbent to a 20 mL solution containing 50 mg/L Cr (VI), shake at room temperature for 6 h, separate by centrifugation, and regenerate the sample using a 0.05 mol/L sodium hydroxide solution (solid–liquid ratio of 1 g/L), shake at room temperature for 6 h, then wash several times with ultrapure water (no less than 3 times, each time with 15 min of ultrasound) until the pH of the wash supernatant is neutral. After centrifugation and filtration, repeat the above steps. Repeat the adsorption–desorption process several times, calculating the adsorption of Cr (VI) by the sample after each adsorption regeneration.

### 3.6. Data Analysis

After repeating each experiment three times, the results were represented as the mean. In instances where there was a difference exceeding 5% between two measurements, additional experiments were carried out. Graph plotting and curve fitting were executed using Origin 9.0. The calculation formulas for removal rate (R) and adsorption capacity (q_t_) can be found in Supplementary Method S2.

## 4. Conclusions

Blue coke is a product of low-temperature coal pyrolysis. Modification with KOH and HNO_3_ generated numerous new pores. LCN exhibits a more diverse pore structure compared to LCK, with an average pore size of 1.30 nm. After being treated with KOH and HNO_3_, the material’s surface functional groups changed. Furthermore, nitric acid modification can remove the inorganic ash content in LCK, resulting in a more developed pore structure. The maximum adsorption capacity, determined using the Langmuir model at 318 K, is 181.962 mg/g. The adsorption of Cr (VI) onto LCN is a spontaneous, endothermic process that increases entropy. Therefore, elevating the temperature is advantageous for Cr (VI) removal by LCN. Adsorption kinetics experiments indicate that the adsorption of Cr (VI) onto LCN involves both physical and chemical adsorption processes. During the adsorption of Cr (VI), electrostatic attraction, electron transfer, electron sharing, and redox reactions occur. These results collectively suggest that LCN holds promise as an adsorbent material for the removal of Cr (VI).

## Figures and Tables

**Table 1:** Comparing this with other adsorbents that are frequently employed for the adsorption of Cr (VI).

Precursor	Activating Agent	Maximal Adsorption Capacity (mg/g)	Reference
Chestnut shells	H_3_PO_4_	85.47	[47]
Corncob	H_3_PO_4_	9.62	[48]
Acacia nilotica (keekar) sawdust	HCl	6.34	[5]
Teakwood sawdust	KOH	72.46	[49]
Corn stalk	KOH	89.5	[7]
Coconut husk	Copper–iron bimetallic nanoparticle-impregnated	173.9	[9]
Medlar	H_3_PO_4_	34.12	[2]
Cucumis melo	H_3_PO_4_	54.28	[2]
Blue coke	KOH	181.96	This

## Data Availability

Data are contained within the article and supplementary materials.

## Supplementary Materials

The following supporting information can be downloaded at: https://www.mdpi.com/article/10.3390/molecules28247986/s1, Method S1: pH Drift Method; Method S2: The removal efficiency (R) and adsorption capacity (q_t_); Supplementary S1: Adsorption Kinetics model; Supplementary S2: Adsorption isotherm model; Supplementary S3: Adsorption thermodynamics; Table S1: BET parameters of LCK and LCN; Table S2: Kinetic parameters of Cr (VI) removal by LCN; Table S3: Adsorption isotherm parameters of Cr (VI) removal by LCN; Table S4: Thermodynamic parameters of Cr (VI)removal by LCN.
