# Supplementary material for: Nitric Acid-Treated Blue Coke-Based Activated Carbon’s Structural Characteristics and Its Application in Hexavalent Chromium-Containing Wastewater Treatment
Source: Molecules. 2023 Dec 7;28(24):7986. doi: 10.3390/molecules28247986 (PMC10745564; doi:10.3390/molecules28247986)
Supplement: Supplementary file 1 [file molecules-28-07986-s001.zip › molecules-2719816-supplementary.pdf]

Nitric acid-treated blue coke-based activated carbon's structural characteristics and its application in hexavalent chromium-containing wastewater treatment

Wencheng Wang[1];Hua Wang[1,2]; Yunxuan Luoyang[1]; Zhang Guotao[1]; Xuchun Gao[1,2]; Jian Li[1]*
[1] College of Chemistry and Chemical Engineering, Yulin University,Yulin City 719000, China;
[2] Shaanxi Provincial Key Laboratory of Clean Utilization of Low-Modified Coal, Yulin University,Yulin City 719000, China;
** Corresponding author at College of Chemistry and Chemical Engineering, Yulin University, Chongwen Road No.51, Yulin City 719000, Shaanxi Province, China*
E-mail address: 99452715@qq.com (Hua Wang).

Method S1. pH Drift Method

In a vial containing 50 mg of the sample, a certain amount of deionized water was added. The pH value was adjusted to a range of 1-10 using potassium hydroxide and hydrochloric acid. Finally, the solution was brought to a total volume of 50 mL. After shaking at room temperature for 24 hours, the pH value was measured. By analyzing the relationship between the final pH value and the initial pH value, the point of zero charge (PZC) was reached.

Method S2. The removal efficiency (R) and adsorption capacity (q_t)

$$R = \frac{(C_0 - C_t)}{C_0} \times 100\% \quad (1)$$

$$q_t = V\frac{(C_0 - C_t)}{m} \quad (2)$$

Parameter Description: C_0 (mg/L) represents the initial concentration of hexavalent chromium; C_t (mg/L) represents the concentration of hexavalent chromium in the solution at time t; V (L) represents the volume of the solution; and m (g) represents the amount of adsorbent added.

Supplementary S1. Adsorption Kinetics model

The equations and parameters for these three kinetic models are presented below:

Pseudo-first-order kinetic model:

$$\ln(q_e - q_t) = \ln q_e - k_1 t \quad (3)$$

Pseudo-second-order kinetic model:

$$\frac{t}{q_t} = \frac{1}{k_2 q_e^2} + \frac{t}{q_e} \quad (4)$$

Intraparticle diffusion model:

$$q_t = k_d t^{0.5} \quad (5)$$

$$q_t = k_d t^{0.5} + C \quad (6)$$

Parameter description: time of adsorption is denoted as 't' (h); 'q_t' (mg · g^{-1}) represents the adsorption capacity at time 't'; 'q_e' (mg · g^{-1}) signifies the equilibrium adsorption capacity. The proportionality coefficients are given as ' k_1(h^{-1})、 k_2 (g · mg^{-1} · h^{-1}), and k_d(mg · g^{1} · h$^{-0.5}$)'. 'C' (mg · g^{-1}) corresponds to the intercept.

Supplementary S2. Adsorption isotherm model

Langmuir model:

$$\frac{C_e}{q_e} = \frac{C_e}{q_m} + \frac{1}{q_m K_1} \quad (7)$$

Freundlich model:

$$\ln q_e = \ln K_F + \frac{1}{n} \ln C_e \quad (8)$$

Parameter Description: K_1（L·mg^{-1}） and K_F（mg$^{1-1/n}$·L$^{1/n}$·g^{-1}） are constants associated with the two isotherm models, which are related to adsorption energy. q_m (mg/g) represents the maximum adsorption capacity, as predicted by the Langmuir model, and 'n' is a constant linked to the Freundlich model, reflecting relative adsorption strength.

Supplementary S3. Adsorption thermodynamics

$$k_d = \frac{q_e}{C_e} \quad (9)$$

$$\Delta G = \Delta H - T\Delta S \quad (10)$$

$$\ln k_d = \frac{\Delta S}{R} - \frac{\Delta H}{RT} \quad (11)$$

Parameter description: R stands for the universal gas constant (8.314 J/(mol·K)), 'T' represents the absolute temperature (K), 'K_d ' represents the equilibrium constant, and 'qe' and 'Ce' are used to denote the equilibrium adsorption capacity and the equilibrium concentration of Cr(VI) at a given temperature.

Table S1. BET parameters of LCK and LCN.

Sample	BET surface area (m^2/g)	Average pore size (nm)	Pore volume (cm^3/g)
LCK	2233.48	1.28	1.4570
LCN	3099.79	1.30	1.9804

Table S2. Kinetic parameters of Cr(VI) removal by LCN.

	Kinetic Model Parameters		
Pseudo-first-order	q_e (mg/ g)	k_1 (h^{-1})	R^2
Cr(VI)	115.416	0.4788	0.7429
Pseudo-second-order	q_e (mg/ g)	k_2 ($g \cdot mg^{-1} \cdot h^{-1}$)	R^2
Cr(VI)	127.961	0.0054	0.9047
Intraparticle diffusion model	C($mg \cdot g^{-1}$)	k_d($mg \cdot g^{1} \cdot h^{-0.5}$)	R^2
Cr(VI)	-	38.8475	1
		22.7664	0.9511
		1.4358	0.9176

Table S3. Adsorption isotherm parameters of Cr(VI) removal by LCN.

Langmuir isotherm				Freundlich Isotherm			
	298K	308K	318K		298K	308K	318K
q_m ($mg \cdot g^{-1}$)	130.869	156.523	181.962	K_F（$mg^{1-1/n} \cdot L^{1/n} \cdot g^{-1}$）	14.7832	16.3088	17.8011
K_l ($L \cdot mg^{-1}$)	66.6389	70.5732	73.5965	n	0.3405	0.3524	0.3618

Langmuir isotherm				Freundlich Isotherm			
R^2	0.9719	0.9561	0.9441	R^2	0.8685	0.8456	0.8323

Table S4. Thermodynamic parameters of Cr(VI)removal by LCN.

			$\Delta G(kJ\cdot mol^{-1}\cdot K^{-1})$		
	$\Delta H(kJ\cdot mol^{-1})$	$\Delta S(kJ\cdot mol^{-1})$	298 K	308 K	318 K
Cr(VI)	25.2546	95.5006	-3.2045	-4.1596	-5.1146
